# Nigrosome 1 imaging in REM sleep behavior disorder and its association with dopaminergic decline

**DOI:** 10.1002/acn3.50962

**Published:** 2019-12-09

**Authors:** Thomas R. Barber, Ludovica Griffanti, Kevin M. Bradley, Daniel R. McGowan, Christine Lo, Clare E. Mackay, Michele T. Hu, Johannes C. Klein

**Affiliations:** ^1^ Oxford Parkinson’s Disease Centre Oxford United Kingdom; ^2^ Nuffield Department of Clinical Neurosciences University of Oxford Oxford United Kingdom; ^3^ Oxford Centre for Human Brain Activity Wellcome Centre for Integrative Neuroimaging Department of Psychiatry University of Oxford Oxford United Kingdom; ^4^ Oxford Centre for Functional MRI of the Brain Wellcome Centre for Integrative Neuroimaging Nuffield Department of Clinical Neurosciences University of Oxford Oxford United Kingdom; ^5^ Department of Radiology Churchill Hospital Oxford United Kingdom; ^6^ Radiation Physics & Protection Department Churchill Hospital Oxford United Kingdom

## Abstract

**Objectives:**

Rapid eye movement sleep behavior disorder (RBD) patients have a high risk of developing a Parkinsonian disorder, offering an opportunity for neuroprotective intervention. Predicting near‐term conversion, however, remains a challenge. Dopamine transporter imaging, while informative, is expensive and not widely available. Here, we investigate the utility of susceptibility‐weighted MRI (SWI) to detect abnormalities of the substantia nigra in RBD, and explore their association with striatal dopaminergic deficits.

**Methods:**

SWI of the substantia nigra was performed in 46 RBD patients, 27 Parkinson’s patients, and 32 control subjects. Dorsal nigral hyperintensity (DNH) was scored by two blinded raters, and separately quantified using a semiautomated process. Forty‐two RBD patients were also imaged with ^123^I‐ioflupane single‐photon emission computed tomography (DaT SPECT/CT).

**Results:**

Consensus visual DNH classification was possible in 87% of participants. 27.5% of RBD patients had lost DNH, compared with 7.7% of control subjects and 96% of Parkinson’s patients. RBD patients lacking DNH had significantly lower putamen dopaminergic SPECT/CT activity compared to RBD patients with DNH present (specific uptake ratios 1.89 vs. 2.33, *P* = 0.002). The mean quantified DNH signal intensity declined in a stepwise pattern, with RBD patients having lower intensity than controls (0.837 vs. 0.877, *P* = 0.01) but higher than PD patients (0.837 vs. 0.765, *P* < 0.001).

**Interpretation:**

Over one quarter of RBD patients have abnormal substantia nigra SWI reminiscent of Parkinson’s, which is associated with a greater dopaminergic deficit. This modality may help enrich neuroprotective trials with early converters.

## Introduction

Parkinson’s disease presents clinically at a time when substantial neurodegeneration in the nigrostriatal system has already occurred.^1^ Identifying early disease during the prodromal phase is therefore important in maximizing the potential of future neuroprotective interventions. Patients with rapid eye movement sleep behavior disorder (RBD) have a long‐term risk exceeding 80% of developing Parkinson’s disease or a related alpha‐synucleinopathy, and therefore, present an opportunity to target the prodromal period.^2^ Due to the long and variable latency, however, near‐term phenoconversion rates are considerably lower, with around 33% of RBD patients on average developing a synucleinopathy within 5 years of RBD diagnosis.^3^ Identification of these highest‐risk patients using disease‐specific markers of neurodegeneration would aid individual prognosis and increase the feasibility of clinical trials aimed at delaying or preventing conversion to motor disease.^4^

Susceptibility‐weighted MRI has emerged recently as a promising tool for evaluating the integrity of the substantia nigra.^5^ In healthy subjects, an area of signal hyperintensity is seen in the dorsal nigra corresponding to nigrosome 1, a nucleus affected early by synuclein‐mediated degeneration.^6^ This dorsal nigral hyperintensity (DNH) is lost in around 98% of patients with Parkinson’s disease.^5^ Three recent studies have indicated that the DNH is absent in a smaller proportion of RBD patients,^7^, ^8^, ^9^ suggesting a potential role in the characterization of prodromal disease progression.

Most studies examining the DNH have used subjective, binary ratings to measure its presence or absence,^10^, ^11^ but the value of SWI imaging as a prodromal progression marker could potentially be enhanced by an objective method of quantifying the DNH signal that reflected the gradually progressive degeneration that occurs in the substantia nigra.

This multimodal neuroimaging study was designed to evaluate the DNH using SWI in a large cohort of polysomnographically proven RBD patients, alongside healthy control subjects and patients with Parkinson’s disease. By performing MRI and dopamine transporter SPECT/CT in parallel, we sought to investigate the relationship between DNH loss and striatal dopaminergic decline in the prodromal phase. We further aimed to develop a semiautomated method of measuring the DNH signal that could objectively quantify the differences between RBD patients, PD patients, and healthy control subjects.

## Methods

### Participants

The study was approved by the local ethical committee and written, informed consent was obtained from all participants. A total of 105 participants were recruited prospectively for the study as volunteers from an existing cohort, described elsewhere.^12^ The initial study population comprised 46 patients with RBD, 27 patients with Parkinson’s disease, and 32 healthy control subjects. RBD was diagnosed by polysomnography according to the International Classification of Sleep Disorders criteria.^13^ In all cases, RBD was considered idiopathic, not secondary to another neurological disorder or medication. RBD patients with evidence of dementia or motor parkinsonism were not eligible for inclusion.

All participants underwent imaging with susceptibility‐weighted MRI and 42 RBD patients were scanned in parallel with ^123^I‐ioflupane SPECT/CT.

### Clinical examination

Participants were examined on the day of imaging by a neurologist using the Movement Disorders Society Unified Parkinson’s Disease Rating Scale (MDS‐UPDRS) part III.^14^ Other clinical motor and nonmotor markers of prodromal parkinsonism were assessed in RBD patients using standard rating scales, described elsewhere.^12^ The probability of prodromal PD was calculated for each RBD patient using the Movement Disorders Society research criteria.^15^

### MRI acquisition and processing

T2*‐weighted images were acquired on a 3T Siemens Trio (Erlangen, Germany) using a 12‐channel receive‐only head coil (gradient‐recalled echo, 256 matrix, voxel size = 0.86 × 0.86 × 1.50 mm^3^, TE/TR = 20 msec/27 msec, flip angle = 15°; 5 min). Axial slices were oriented parallel to the plane between the anterior and posterior commissures. Phase images were high‐pass filtered using a 50 × 50 window in Fourier space to remove macroscopic phase artifacts. This window size was selected empirically to suppress artifacts in the midbrain caused by nearby aerated structures. Paramagnetic phase components only were taken to the fourth power and then multiplied with the magnitude images to give the final susceptibility‐weighted images.

T1‐weighted structural MRI was acquired to facilitate the registration of SWI images to standard space, with acquisition parameters as follows: 3T Siemens Trio (Erlangen, Germany), 12‐channel receive‐only head coil, MPRAGE, TE/TR/TI = 4.7 msec/2040 msec/900 ms; 192 axial slices; isotropic voxel size 1 mm^3^; 6 min.

### SPECT/CT acquisition and analysis

The protocol used for SPECT/CT acquisition and image processing was performed as previously described^16^. SPECT/CT data were analyzed using BRASS software (HERMES Medical Solutions AB, Stockholm). Reconstructed images for each patient were registered to a standard template including regions of interest (ROIs) for the caudate and putamen on each side. Uptake ratios were calculated for these ROIs using a standard reference region. Additionally, each SPECT/CT scan was reported descriptively in a standard clinical manner by an experienced nuclear medicine radiologist (KMB) who was blinded to all clinical and MRI data except for the participants’ age, gender, and presence of RBD. The descriptive reports were categorized as either normal, abnormal, or borderline.

### MRI analysis

**Figure 1:**
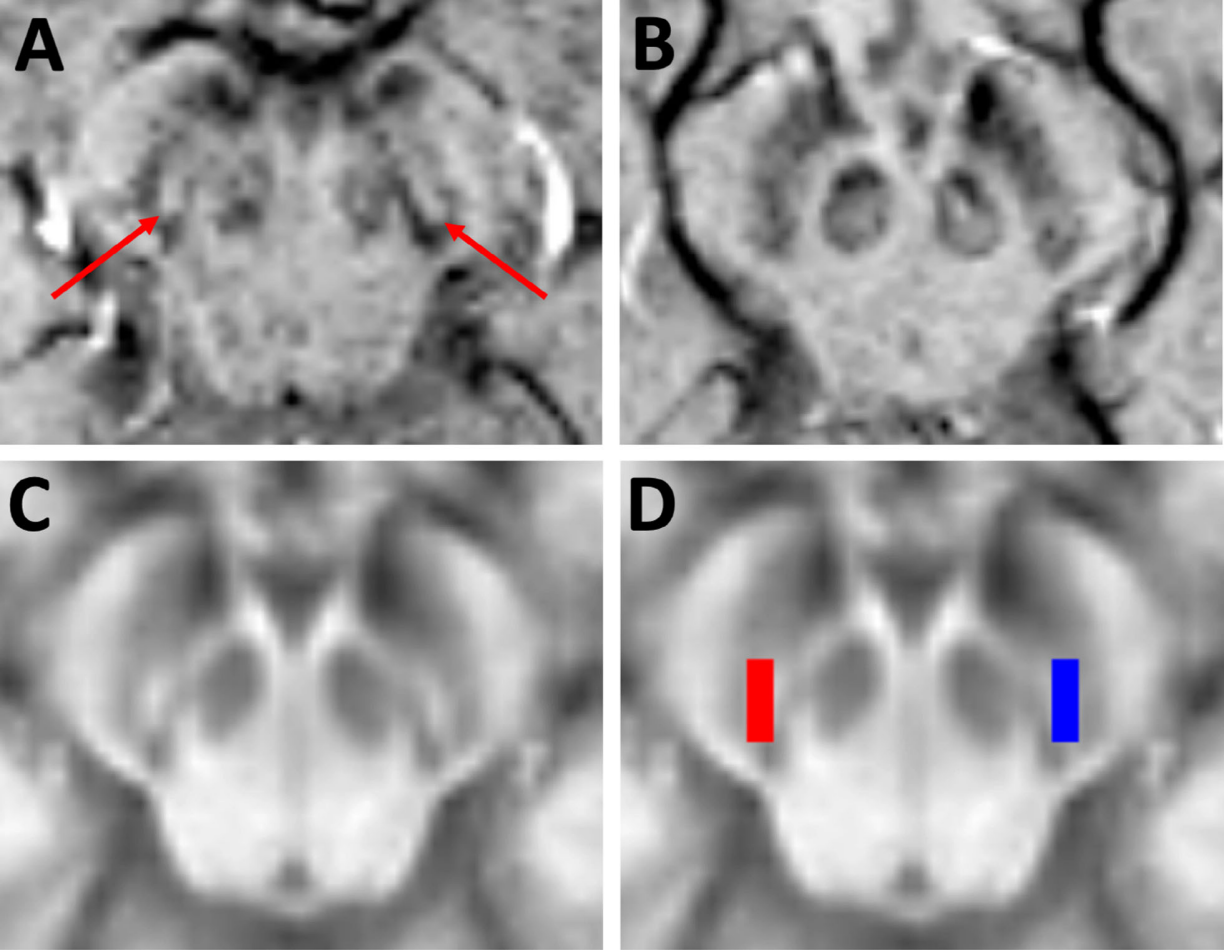
(A and B) Example SWI images at the level of the substantia nigra from a control participant (A) and a patient with Parkinson’s disease (B). The dorsal nigral hyperintensity can be seen in the control participant (red arrows), but is absent in the patient with Parkinson’s disease. (C and D) images used in quantification of the DNH signal. (C) A template image showing the average normalized SWI intensity across 32 healthy control subjects. (D) regions of interest from the left (blue) and right (red) DNH were used to calculate the mean DNH signal intensity in each participant. Abbreviations: SWI, susceptibility‐weighted imaging; DNH, dorsal nigral hyperintensity.

For the subjective ratings of the dorsal nigral hyperintensity (DNH), SWI images from all 105 subjects were first anonymized and compiled into a single 4D file in random order by an investigator independent from the two raters. Assessments were then performed independently by two separate raters blinded to all clinical details including subject status (i.e., control, PD, or RBD), using a method based on that described by Schwarz et al.^10^ Rater 1 was a clinical neurologist with expertize in neuroimaging. Rater 2 was a research scientist specializing in brain MRI analysis. Briefly: for each subject the DNH was scored as present, absent or uncertain for the left and right substantia nigra, respectively (Fig. 1A and B). Using these data the scans were classified into two categories as follows: (1) bilateral presence of the DNH, or unilateral presence with the contralateral side uncertain, was considered normal and is referred to as “DNH present;” (2) definite absence of the DNH unilaterally or bilaterally was considered abnormal and is referred to as “DNH absent.” In cases where one rater classed the DNH as uncertain bilaterally and the other rater gave a definite classification, the definite classification was used. Cases where both raters considered the images uncertain bilaterally were classed as “nondiagnostic.” Where there was disagreement in outcome between the two raters and one rater’s scoring included “uncertain” for one side, a consensus rating was sought by overruling the uncertain score (e.g., if left/right was scored by one rater as “present/absent” [i.e., abnormal] and by the other rater as “present/uncertain” [i.e., normal], the final rating used would be “present/absent”).

For the quantification of DNH signal, SWI images of all participants were first nonlinearly registered to Montreal Neurological Institute (MNI) standard space via T1‐weighted images. Registered SWI images were then normalized to the background image intensity for each individual by dividing the whole image by the mean intensity of a region of interest in the brainstem parenchyma remote from the substantia nigra (Fig. S1). A single, study‐specific template image was created in MNI space using the mean values from the 32 healthy control subjects to determine the normal average location of the DNH (Fig. 1C). Using this template as a guide, a single, standard space region of interest was drawn manually in the area of the DNH on each side (Fig. 1D). The mean intensity within this template nigrosome ROI was then extracted from each individual subject’s normalized, standard space images. Values presented are the mean of left and right DNH ROIs for each subject.

### Statistical methods

Pairwise comparisons between groups were performed using a chi‐square test for dichotomized variables, and an independent samples t‐test for continuous variables. Comparison between contralateral and ipsilateral values within RBD patients was performed using a paired samples t‐test. Correlations were calculated as Pearson coefficients. All statistical analyses were performed using SPSS (IBM) version 25.

## Results

### Visual DNH assessment

Table 1 shows a breakdown of all subjects included in the initial study population. Eight of 105 participants (four RBDs, two PDs, and two controls) had SWI images that were of inadequate quality for DNH assessment due to image artifacts, and were excluded from all analyses. Baseline demographic and clinical variables for the 97 included participants are shown in Table 2.

**Table 1:** Breakdown of the number of subjects included at each stage of the analysis.

		Controls	PD patients	All RBD patients	RBD with parallel DaT SPECT/CT imaging
(A)	Number of subjects imaged with SWI	32	27	46	42
(B)	Number of subjects excluding those with motion‐degraded SWI images (subjects included in automated DNH quantification)	30	25	42	38
(C)	Number of subjects with diagnostic imaging and consensus visual DNH ratings (subjects included in visual DNH outcome analysis)	26	25	40	36

Abbreviations: SWI, susceptibility‐weighted imaging; PD, Parkinson’s disease; RBD, rapid eye movement sleep behaviour disorder; DaT SPECT/CT, Dopamine transporter single‐photon emission computed tomography with computed tomography‐based attenuation correction; DNH, dorsal nigral hyperintensity.

**Table 2:** Baseline demographic and clinical variables.

	Controls	RBD patients	PD patients	*P*‐value
	*N* = 30	*N* = 42	*N* = 25	
Age, years, mean (SD)	69.1 (8.15)	64.8 (8.03)	64.8 (10.12)	Con vs RBD: 0.03
				Con vs PD: 0.08
				RBD vs PD: 0.97
Sex (% male)	80%	98%	60%	Con vs RBD: 0.01
				Con vs PD: 0.10
				RBD vs PD: <0.001
Disease duration, years, mean (SD)^1^	n/a	2.5 (2.44)	5.2 (2.31)	n/a
UPDRS III score, mean (SD)	2.7 (2.07)	5.6 (3.68)	31.9 (14.28)	Con vs RBD: <0.001
				Con vs PD: <0.001
				RBD vs PD: <0.001

Abbreviations: RBD, rapid eye movement sleep behaviour disorder; PD, Parkinson’s disease; Con, controls; UPDRS, Unified Parkinson’s Disease Rating Scale.

1 For RBD patients, time since RBD diagnosis by polysomnography; for PD patients, time since clinical diagnosis.

One included scan was considered nondiagnostic by both raters in visual assessment of the DNH. Of the remaining 96 scans, there was concordance between the two raters in 89 (93%). In the seven scans in which there was initial disagreement, a consensus rating was achieved in two, leaving five cases that could not be resolved. These five cases with inter‐rater disagreement comprised four control subjects and one RBD patient. The results of visual DNH assessments for the 91 subjects with consensus ratings are shown in Table 3.

**Table 3:** Results of binary visual assessments of the dorsal nigral hyperintensity.

	Controls	RBD patients	PD patients
	*N* = 26	*N* = 40	*N* = 25
Number with DNH present (% of patient group)	24 (92.3%)	29 (72.5%)	1 (4.0%)
Number with DNH absent (% of patient group)	2 (7.7%)	11 (27.5%)	24 (96.0%)

Abbreviations: RBD, rapid eye movement sleep behaviour disorder; PD, Parkinson’s disease; DNH, dorsal nigral hyperintensity.

Excellent discrimination accuracy was observed between healthy controls and patients with Parkinson’s disease, with the absence of DNH achieving a sensitivity of 96%, specificity of 92%, positive predictive value of 92% and negative predictive value of 96%. Among RBD patients with rateable images, 11 of 40 (27.5%) had absence of the DNH, while 29 (72.5%) had DNH present. The rate of DNH absence in RBD patients was significantly higher than controls (27.5% vs. 7.7%, *P* = 0.048) and significantly lower than PD patients (27.5% vs. 96.0%, *P* < 0.001).

Unilateral abnormalities were more common in RBD than PD patients, perhaps suggesting an intermediate pattern of abnormality. Of the 11 RBD patients with DNH absence, seven (64%) had retained DNH presence on one side, whereas in PD patients with DNH absence, only 2 of 24 cases (8%) had unilateral DNH presence.

### Association of DNH loss with prodromal markers in RBD

Table 4 shows the scores across a range of prodromal markers in RBD patients, grouped according to the presence or absence of DNH. Among individual markers, none were more severely affected in RBD patients who had lost DNH. However, when markers were combined using the MDS research criteria for prodromal Parkinson’s disease, RBD patients with DNH absence had a significantly higher probability of prodromal Parkinson’s than those with DNH presence, suggesting that DNH loss in RBD is associated with an increased risk of future Parkinson’s disease.

**Table 4:** Clinical parkinsonian features compared in RBD patients according to the presence or absence of the dorsal nigral hyperintensity (DNH).

	RBD DNH present	RBD DNH absent	*P* value
	*N* = 29	*N* = 11	
MDS‐UPDRS III	5.14 (3.57)	5.73 (3.74)	0.65
Purdue Pegboard score	32.1 (5.61)	34.0 (3.23)	0.30
Olfaction (Sniffin Sticks score)	8.2 (3.51)	6.6 (3.86)	0.25
Postural systolic blood pressure change (mm Hg)	−7.7 (13.9)	−1.4 (14.1)	0.24
MoCA score	25.4 (3.18)	26.0 (2.28)	0.57
Epworth sleepiness score	7.4 (4.35)	5.8 (5.69)	0.37
Beck depression inventory score	10.7 (9.73)	6.3 (4.59)	0.07
Anxiety score (HADS)	7.1 (4.64)	3.6 (3.93)	0.04
Apathy severity (LARS)	−19.8 (7.52)	−23.6 (4.57)	0.13
MDS prodromal PD probability, %	82.2 (19.6)	95.2 (9.19)	0.01

Abbreviations: MDS, Movement Disorders Society; UPDRS, Unified Parkinson’s Disease Rating Scale; MoCA, Montreal Cognitive Assessment; HADS, Hospital Anxiety and Depression Scale; LARS, Lille Apathy Rating Scale.

### Comparison of SWI and DaT SPECT/CT imaging

Of the 42 RBD patients with parallel SWI and DaT SPECT/CT imaging, 36 had diagnostic SWI imaging with consensus DNH ratings (Table 1). Nineteen (53%) of these patients had normal DaT SPECT/CT imaging as rated by the blinded radiologist, while 14 (39%) had abnormal imaging and three (8%) were classed as borderline. The results of the SWI DNH assessments according to these categorical DaT SPECT/CT outcomes are shown in Table 5. There was good agreement between imaging modalities for the 19 RBD patients with normal dopaminergic imaging: 17 (89%) of these individuals had retained the presence of the DNH. There was less concordance in the 14 RBD patients with abnormal dopaminergic imaging: six (43%) of these patients had DNH present, while eight (57%) had loss of the DNH. One of the three RBD patients with borderline dopaminergic imaging had loss of the DNH and the other two had DNH present.

**Table 5:** Comparison of DNH assessments and DaT SPECT/CT outcomes in RBD patients.

RBD patients	DaT SPECT/CT	DaT SPECT/CT	DaT SPECT/CT
*N* = 36	Normal	Abnormal	Borderline
DNH present	17	6	2
DNH absent	2	8	1

Abbreviations: RBD, rapid eye movement sleep behaviour disorder; DNH, dorsal nigral hyperintensity; DaT SPECT/CT, dopamine transporter single‐photon emission computed tomography with CT attenuation correction.

**Figure 2:**
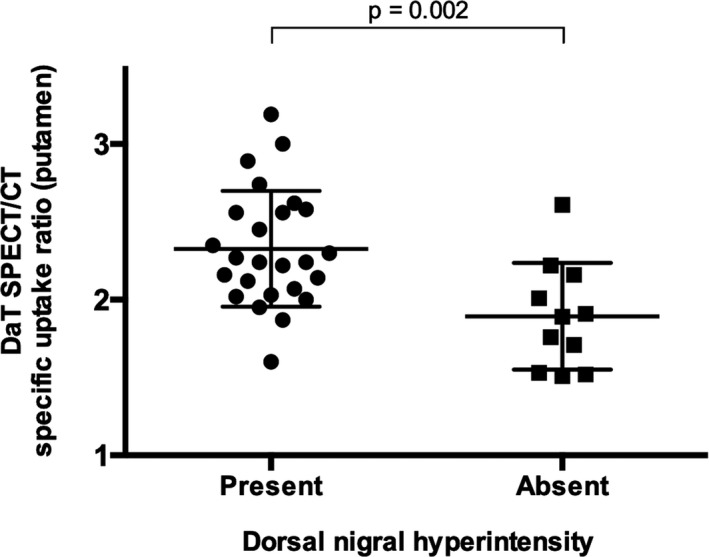
Dopaminergic SPECT/CT signal from the putamen in RBD patients, grouped according to the presence or absence of dorsal nigral hyperintensity on SWI imaging. RBD patients with loss of DNH had significantly lower dopaminergic signal than those with DNH present. Abbreviations: SWI, susceptibility‐weighted imaging; DNH, dorsal nigral hyperintensity; RBD, rapid eye movement sleep behavior disorder; SPECT/CT, single‐photon emission computed tomography with CT attenuation correction.

Figure 2 shows DaT SPECT/CT ratios extracted from the putamen (mean of left and right) of RBD patients grouped according to the presence or absence of DNH on SWI imaging. Those with absence of the DNH had significantly lower DaT SPECT/CT signal in the putamen compared to those with the DNH present (mean putamen uptake ratios 1.89 vs. 2.33, *P* = 0.002).

In the seven RBD patients with unilateral loss of the DNH, dopaminergic signal in the putamen ipsilateral to the DNH loss was significantly lower than in the contralateral putamen (mean putamen ratios 1.72 ipsilateral vs. 1.87 contralateral, *P* = 0.01).

### Quantification of DNH signal intensity

**Figure 3:**
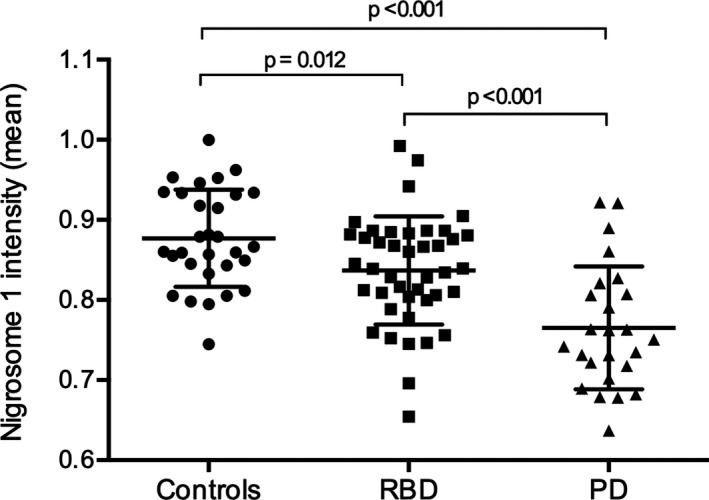
Mean DNH signal intensities (reported as a proportion of background intensity) calculated from SWI show a stepwise decline across the three groups of participants. Pairwise comparisons show significant differences between all three groups. Abbreviations: SWI, susceptibility‐weighted imaging; DNH, dorsal nigral hyperintensity.

The normalized mean intensity values extracted from the region of the DNH are shown in Figure 3, grouped according to patient category and excluding only the eight subjects with artifact‐degraded images (N.B. the five subjects with inter‐rater discordance and the one subject with nondiagnostic imaging by visual rating are included). Significant differences were observed between the means in all three pairwise comparisons, with a stepwise decline in signal intensity from controls to RBD patients and from RBD patients to PD patients. In a receiver operating characteristic (ROC) analysis, the mean intensity of the DNH region separated PD patients from controls with an area under the curve (AUC) of 0.86, with a cutoff value of 0.823 giving an optimum combination of sensitivity (84%) and specificity (80%). Among RBD patients, 19 of 42 (45%) had DNH signal intensity below this cutoff level.

The mean DNH intensity did not correlate with UPDRS III scores in either RBD patients (*r* = 0.15, *P* = 0.33) or PD patients (*r* = 0.10, *P* = 0.65). There was no significant difference in DNH intensity between RBD patients with normal versus abnormal dopaminergic imaging (0.836 vs. 0.826, *P* = 0.66), nor any direct correlation between mean DNH intensity and mean DaT SPECT/CT signal in the putamen (*r* = −0.24, *P* = 0.15).

## Discussion

Our study demonstrates that around a quarter of patients with idiopathic RBD have loss of the dorsal nigral hyperintensity (DNH) on SWI imaging, a pattern of abnormality that occurs in established Parkinson’s disease and indicates degeneration affecting nigrosome 1. Postmortem studies have shown dopaminergic neuronal loss in PD to be highest in nigrosome 1 compared to other substantia nigra regions, with a maximal 98% loss seen.^17^ A subsequent pathoanatomical correlation using in vivo 7T MRI confirmed that the loss of imaging hyperintensity seen in nigrosome 1 corresponds to the loss of neuromelanin‐containing cells seen in PD patients.^6^ Our study therefore provides important *in vivo* evidence of prodromal disease in the substantia nigra and raises the possibility that SWI may be of value in the risk stratification in RBD patients. The observation that RBD patients with loss of the DNH have reduced dopamine transporter binding in the putamen compared to those with the DNH present is important evidence that this SWI sign is indicative of nigrostriatal integrity. This is supported by the finding of a higher probability of prodromal PD, calculated using the MDS research criteria, in patients with DNH loss.

Our data add to a growing body of neuroimaging evidence from populations at risk of Parkinsonian disorders indicating that degeneration‐related changes are detectible early in the disease process.^18^ Loss of the dorsal nigral hyperintensity has also been reported in asymptomatic LRRK2 mutation carriers, suggesting that this marker may be applicable to preclinical and prodromal disease beyond those with RBD.^19^ The finding that nigral R2* values in LRRK2 carriers are increased relative to controls supports the idea that iron deposition may be the mechanism underlying the imaging phenotype.^20^ Other MRI modalities to have demonstrated disease‐related changes in the nigrostriatal system in RBD patients include neuromelanin‐sensitive imaging^21^ and resting state functional connectivity,^22^ while the evidence of dopaminergic dysfunction is now firmly established.^23^ Neuroimaging therefore holds great promise for the identification of individuals who might benefit the most from targeted neuroprotective interventions.

Comparison of our SWI outcomes with three smaller studies that have previously investigated loss of the DNH in RBD patients reveals some important differences: all three studies reported higher rates of DNH loss than we have observed, as well as a higher proportion of bilateral abnormalities (Table 6).^7^, ^8^, ^9^ Methodological variations including differences in imaging protocol, rating strategy and rater experience may have influenced outcomes, and it is possible that the effect of these is greater in RBD patients than in controls or PD patients, where the presence of borderline cases may increase the ambiguity of binary assessment.

**Table 6:** Rates of DNH absence in published RBD studies.

Study	Number of RBD patients with adequate imaging	Mean age of RBD patients, years	Mean RBD duration, years	Proportion with absent DNH on SWI	Proportion of abnormal cases with unilateral DNH absence
De Marzi et al., 2016^6^	13	68.9	5.3	10/13 (77%)	1/10 (10%)
Frosini et al., 2017^7^	16	69.3	6.3	9/16 (60%)	2/9 (22%)
Bae et al., 2018^8^	18	70.5	5.9	11/18 (61%)	2/11 (18%)
This study	40	64.5	2.5	11/40 (28%)	7/11 (64%)

Abbreviations: RBD, rapid eye movement sleep behaviour disorder; SWI, susceptibility‐weighted imaging; DNH, dorsal nigral hyperintensity.

However, it is also possible that our patients are on average at an earlier prodromal stage. The long and variable latency to phenoconversion in RBD patients means that the small cohorts described previously could be easily influenced by a few individuals who are near to conversion. One study, for example, reported DNH absence in 11 of 18 RBD patients.^9^ However, they also observed conversion to a neurodegenerative disorder in 5 of 18 RBD patients (28%) after an average 18‐month follow‐up period. This is much higher than would be expected from the overall average conversion rate of 6% per year in RBD,^24^ and suggests that their RBD patients were at a relatively advanced prodromal stage.

In the absence of longitudinal data, we cannot yet say if our patients were at an earlier stage at the time of their imaging; although they were younger and had a shorter RBD disease duration than other studies, neither of these are reliable indicators of prodromal staging.

If it were the case, however, that more advanced prodromal cases had higher rates of DNH loss, it would suggest that this marker could develop as patients progress through the stage of prodromal disease after RBD diagnosis. Such sensitivity to disease progression would be an important characteristic, and in contrast to substantia nigra ultrasound, another binary risk marker, which does not show change overtime.^25^

There was partial discrepancy in findings between the dichotomised outcomes of SWI and DaT SPECT/CT in our study. While we observed DNH loss in only 28% of our RBD patients, the overall rate of DaT SPECT/CT abnormality was higher at 39%, a figure broadly in line with other studies indicating that 40–50% of RBD patients have evidence of a dopaminergic deficit.^26^, ^27^ Interestingly, 89% of those with normal dopaminergic imaging also had normal SWI imaging, whereas only 57% of RBD patients with a dopaminergic deficit had absence of the DNH.

One potential explanation for this would be that DNH loss may occur at a later stage of nigrostriatal degeneration than dopamine transporter deficit, again in keeping with a relatively early stage of pathology in our cohort. The retained presence of the DNH alongside abnormal dopaminergic imaging would be consistent with the proposed “dying‐back” pattern of nigrostriatal axonal degeneration, in which synaptic dysfunction precedes loss of the nigral cell bodies.^28^

The hypothesis that DNH loss is a later prodromal sign than DaT abnormality remains to be tested by long‐term follow‐up of the patients in this study. However, if this was the case, it would have important implications for the risk stratification of RBD patients, with perhaps greater potential for the enrichment of near‐term converters using SWI. This would be advantageous in the development of prodromal cohorts for neuroprotective clinical trials in which conversion to an overt synucleinopathy were the primary outcome measure, and could rationalize ionizing radiation use by targeting only those study participants for DaT scan who have a high probability of a change in dopaminergic transmission.

It is notable that the degree of discordance in outcomes that we observed between SWI and DaT SPECT/CT is greater than has been reported previously.^8^, ^9^ This is mainly due to the proportion of our RBD patients with DNH present who had abnormal DaT SPECT/CT outcomes. In contrast, the high positive predictive value of DNH loss for DaT SPECT abnormality is a consistent finding across all studies: when our data are combined with these two previous studies, more than 90% of RBD patients with loss of DNH have had abnormal dopaminergic imaging.^8^, ^9^ This makes DNH a potentially useful stratification tool for clinical trial recruitment, where a high positive predictive value for underlying disease would be more important than a high sensitivity.

Despite the promise of nigral SWI as a biomarker, it is limited in its observer rating system by a degree of subjectivity, inter‐rater discrepancy, and a binary outcome measure that lacks quantification. Although this method performs well enough to accurately discriminate Parkinson’s patients from controls, a binary outcome may be more problematic in prodromal patients where there are expected to be many more borderline cases. The simple method of signal quantification that we have used in this study attempts to overcome some of these limitations.

The overall pattern of results obtained using this method matched that of the binary ratings, with a stepwise decline in dorsal nigral intensity from controls to RBD patients and from RBD patients to PD patients. The objective DNH intensity measure performed well in separating PD patients from controls and although this discrimination was slightly less accurate than visual ratings, importantly it included subjects with nondiagnostic visual ratings. A possible explanation for this is that automated signal quantification will not distinguish between high signal from nigrosome 1 and high signal caused by artifacts. Imperfect registration may also lead to a limited amount of bright signal from outside the nigra being included inadvertently in some cases. It is possible, therefore, that a combination of visual assessment and signal quantification would be required to maximize accuracy.

As with the binary observer ratings, RBD patients were more similar to controls than to PD patients, but significantly different from both. Interestingly, when applying the optimal DNH intensity cutoff from the PD versus controls ROC analysis to RBD patients, a higher percentage was classified as abnormal (45%) than with the subjective ratings (28%). It remains to be seen with longitudinal follow‐up whether objective SWI measurements, and their change overtime, outperform binary ratings in delineating disease stage. However, the intermediate spread of values observed in RBD patients in cross‐sectional assessment suggests that the decline in nigrosome 1 signal may be a gradual process.

The fact that we did not observe a correlation between motor severity and DNH signal intensity may be accounted for by a floor effect in PD patients. The majority had bilateral complete loss of the DNH and it is likely that the low signal remaining in the dorsolateral nigra (Fig. 1B) is insensitive to further change. In RBD patients this was also an unsurprising finding, since motoric disease is largely absent, UPDRS III scores are low, and no other nigrostriatal imaging modality has demonstrated any reproducible correlation with UPDRS III scores in RBD patients.^29^

The fact that we did not observe any direct correlation between the SWI and DaT SPECT/CT measurements suggests that they describe distinct aspects of nigrostriatal integrity. It is possible, therefore, that a combination of the two modalities may be more informative than either test alone. Further development of multimodal imaging assessments will be an important avenue of future research, including the combination of SWI with measures from other MRI sequences such as neuromelanin‐sensitive and diffusion‐weighted MRI.^21^ Quick and objective means of signal quantification will be crucial in such large multimodal models.

The large number of patients with polysomnographically proven RBD included in this study, as well as the multimodal imaging approach and semiautomated SWI analysis are major strengths. Nevertheless, we acknowledge some important limitations. First, the three groups are not precisely matched for age and sex, and although previous studies have shown that age and sex do not significantly affect binary assessments of the DNH,^10^, ^11^ we cannot rule out an element of bias as a result of incomplete matching. Second, ethical approval restrictions prevented us from obtaining DaT SPECT/CT data in our control participants. While this did not affect the principal aim of comparing dopaminergic signal between RBD patients with and without DNH loss, it did mean that our dichotomization of normal and abnormal DaT SPECT/CT outcomes was not made with reference to our control population. However, we believe that the subjective evaluation by an experienced, blinded, clinical radiologist is an equally valid method of assessment.

In conclusion, SWI imaging of the DNH is a promising biomarker of prodromal neurodegeneration in RBD patients, who exhibit an intermediate rate of abnormality between PD patients and healthy controls. RBD patients with loss of DNH have higher rates of dopaminergic deficits compared to those without, and consequently may be at higher risk of developing a Parkinsonian disorder in the near‐term. This finding suggests that SWI may be a viable tool with which to enrich prodromal cohorts for neuroprotective trials. Longitudinal assessments will determine the predictive value of SWI imaging as well as the ability of DNH quantification to detect meaningful nigral degeneration overtime.

## Author Contributions

All authors were involved in study design and conception, and editing and revising manuscript. Barber, Griffanti, Bradley, McGowan, and Lo were involved in data acquisition. Barber, Griffanti, Bradley, and Klein were involved in data analysis. Barber, Griffanti, and Klein were involved in drafting of manuscript.

## Conflict of Interest

None.

## Supporting information


**Figure S1.** The region of interest marked in red was used to define the background brainstem signal intensity in each subject in order to normalize the signal intensities extracted from the substantia nigra.
