# Supplementary figures and images for: Nigrosome 1 imaging in REM sleep behavior disorder and its association with dopaminergic decline
Source: Ann Clin Transl Neurol. 2019 Dec 9;7(1):26–35. doi: 10.1002/acn3.50962 (PMC6952317; doi:10.1002/acn3.50962)

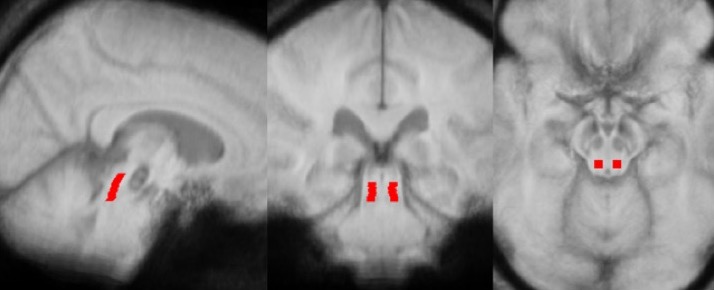

Supplement: Supplementary file 1 — Figure S1. The region of interest marked in red was used to define the background brainstem signal intensity in each subject in order to normalize the signal intensities extracted from the substantia nigra. [file ACN3-7-26-s001.tiff]
